# Real-World Outcomes of First-Line Palbociclib Plus Endocrine Therapy for HR+/HER2− Metastatic Breast Cancer in Japan: A Single-Center Retrospective Study

**DOI:** 10.32604/or.2025.073891

**Published:** 2025-12-30

**Authors:** Keiko Yanagihara, Masato Yoshida, Kensaku Awaji, Tamami Yamakawa, Sena Kato, Miki Tamura, Koji Nagata

**Affiliations:** 1 Department of Breast Surgery and Oncology, Nippon Medical School Tama-Nagayama Hospital, 1-7-1 Nagayama, Tama-shi, Tokyo, 206-8512, Japan; 2 Department of Pharmacy, Nippon Medical School Tama-Nagayama Hospital, 1-7-1 Nagayama, Tama-shi, Tokyo, 206-8512, Japan; 3 Department of Pathology, Nippon Medical School Tama-Nagayama Hospital, 1-7-1 Nagayama, Tama-shi, Tokyo, 206-8512, Japan

**Keywords:** Metastatic breast cancer, cyclin-dependent kinase 4/6 (CDK4/6) inhibitors, real-world evidence, hormone receptor-positive, human epidermal growth factor receptor 2-negative breast cancer

## Abstract

**Background:**

Cyclin-dependent kinase 4/6 (CDK4/6) inhibitors have transformed the management of hormone receptor–positive/HER2–negative (HR+/HER2–) advanced breast cancer, yet evidence for elderly or poor-performance patients remains limited. This study examined real-world outcomes of palbociclib plus endocrine therapy in Asian patients, with additional subgroup analyses by age and performance status.

**Methods:**

We retrospectively analyzed 46 consecutive Asian patients with recurrent or *de novo* HR+/HER2− breast cancer treated with first-line palbociclib plus ET between April 2021 and March 2025. The primary endpoint was progression-free survival (PFS). Secondary endpoints included overall response rate (ORR), disease control rate (DCR), and safety. Subgroup analyses were performed by age (<70 vs. ≥70 years) and performance status (PS; 0–1 vs. 2–3).

**Results:**

The median PFS was 26.6 months (range, 1.4–69.5). Stratified by age, median PFS was 26.9 months in patients <70 years and 26.2 months in those ≥70 years (*p* = 0.760). By PS, PFS was 26.9 months for PS 0–1 and 17.8 months for PS 2–3 (*p* = 0.099). ORR was 60.9% and DCR 93.5%; notably, all PS 2–3 patients achieved disease control. Hematologic toxicities were common, with neutropenia (80.4%) and leukopenia (86.7%) predominating, but grade ≥ 3 anemia was rare (2.2%). Elderly patients experienced anemia more frequently, while overall toxicity remained manageable. Dose reductions occurred in 47.8% without loss of efficacy.

**Conclusions:**

In routine Japanese practice, palbociclib plus ET provided prolonged PFS and high disease control consistent with pivotal trials and international real-world evidence. Importantly, elderly patients tolerated treatment well, and selected PS 2–3 patients also derived clinical benefit. These findings indicate that neither age nor PS alone should preclude the use of palbociclib in carefully monitored real-world patients.

## 1 Introduction

Breast cancer is the most frequently diagnosed malignancy among women worldwide and remains one of the leading causes of cancer-related mortality [1]. In 2020, more than two million new cases were reported globally, with nearly 700,000 deaths. Although survival has improved with early detection and systemic therapy [1], advanced breast cancer continues to present a substantial clinical challenge.

Among breast cancer subtypes, hormone receptor-positive, human epidermal growth factor receptor 2-negative (HR+/HER2−) disease is the most common, representing more than two-thirds of cases [1]. The relatively indolent natural history of this subtype allows for long-term control with endocrine therapy (ET), which has been the cornerstone of treatment for decades. However, resistance to ET inevitably develops, leading to progression and mortality [2].

The development of CDK4/6 inhibitors revolutionized the management of HR+/HER2− advanced breast cancer. The PALOMA trials with palbociclib showed marked improvement in PFS over endocrine therapy alone [3–5]. Similar results were observed in the MONALEESA trials with ribociclib, which also demonstrated OS benefits [6–8]. Abemaciclib trials (MONARCH-2 and -3) further confirmed these findings, establishing CDK4/6 inhibitors as the standard first-line treatment for HR+/HER2− disease [9,10].

Despite these successes, pivotal trials enrolled relatively younger, fitter patients. Median ages were 56–63 years, and nearly all participants had ECOG performance status (PS) 0–1 [11–13]. Patients aged 70 or older and those with poor PS (≥2) were under-represented [11]. Yet in daily practice, especially in aging societies like Japan, such patients constitute a significant proportion of cases. This discrepancy creates uncertainty regarding the generalizability of trial results.

Real-world evidence (RWE) from Western countries has confirmed the efficacy of palbociclib in elderly patients [14–16]. Safety analyses also indicate manageable toxicity with appropriate dose modifications [17,18]. However, evidence from Asia remains limited, a region with a relatively smaller body stature, and subgroup analyses for elderly or poor-PS patients are scarce [19–21]. Performance status is particularly important: although patients with PS 2–3 are often excluded from trials, some observational data suggest that selected poor-PS patients may still benefit [22].

Therefore, we conducted a retrospective cohort study of Asian patients treated with first-line palbociclib plus ET for recurrent or *de novo* HR+/HER2− breast cancer. We aimed to evaluate efficacy and safety, with a focus on age and PS subgroups. We also compared our results with pivotal trials, international RWE, and clinical guidelines, and explored the implications for treating vulnerable patient populations.

## 2 Patients and Methods

### 2.1 Study Design and Eligibility

This was a single-center retrospective study conducted at Nippon Medical School Tama-Nagayama Hospital in Japan. Eligible patients were women with histologically confirmed HR+/HER2− breast cancer who initiated palbociclib plus endocrine therapy (ET) as first-line systemic treatment for recurrent or *de novo* metastatic disease between April 2021 and March 2025. Patients who had received prior systemic therapy in the metastatic setting were excluded.

The study protocol was reviewed and approved by the Institutional Review Board of Nippon Medical School Tama-Nagayama Hospital (Approval No. M-2025-350), and the requirement for individual informed consent was waived because of the retrospective nature of the study. Patients were provided the opportunity to opt out via public notice in accordance with institutional and national ethical guidelines.

### 2.2 Treatment Regimen

Palbociclib was given orally at 125 mg once daily for 21 consecutive days, followed by 7 days off (28-day cycle). All patients started at the full dose. Dose reductions to 100 mg or 75 mg were implemented for recurrent grade ≥ 3 hematologic toxicities or intolerable non-hematologic adverse events (AEs). Temporary dose interruptions were allowed until recovery. Concomitant ET partners were aromatase inhibitors (letrozole or anastrozole) or fulvestrant, chosen at the physician’s discretion. Premenopausal women also received a gonadotropin-releasing hormone (GnRH) agonist.

### 2.3 Clinical Assessments

Baseline demographics, tumor characteristics, and treatment histories were collected. Image evaluation was performed as appropriate. Tumor responses were evaluated according to the Response Evaluation Criteria in Solid Tumors (RECIST), version 1.1 [23]. Hematological tests were obtained at baseline and at least once per cycle. AEs were graded according to Common Terminology Criteria for Adverse Events (CTCAE), version 5.0 [24].

The primary endpoint was PFS, defined as the time from initiation of palbociclib to disease progression or death. Secondary endpoints included overall response rate (ORR), disease control rate (DCR), safety, dose modifications, and treatment discontinuations. Exploratory subgroup analyses were conducted by age (<70 vs. ≥70 years) and PS (0–1 vs. 2–3).

### 2.4 Statistical Analysis

Descriptive statistics summarized baseline data and AEs. Kaplan–Meier methods estimated PFS, with comparisons by log-rank tests. Cox proportional hazards analysis was performed to estimate hazard ratios (HRs) and 95% confidence intervals (CIs) for progression-free survival (PFS). The primary comparisons were age (<70 vs. ≥70 years) and performance status (PS 0–1 vs. PS 2–3). To avoid model overfitting due to the limited number of events, the number of covariates in multivariate models was restricted to two or three clinically relevant variables (e.g., *de novo* metastasis, serosal involvement). Ties were handled using the Efron method, and two-sided *p*-values <0.05 were considered statistically significant. These analyses were performed using Python (statsmodels package). Fisher’s exact test or chi-square test was used to evaluate categorical differences in responses and AEs. Statistical analyses were performed using R version 4.3.1 (R Foundation for Statistical Computing, Vienna, Austria) [25].

## 3 Result

### 3.1 Patient Characteristics

Forty-six patients with hormone receptor-positive, HER2− negative recurrent or metastatic breast cancer were included in this study. Baseline characteristics of the entire cohort and subgroup distribution based on age and PS are summarized in Table 1.

**Table 1:** Characteristics of patients (overall, by age group, and by performance status)

Patient subgroup	All patients (*n* = 46)	<70 yrs (*n* = 22)	≥70 yrs (*n* = 24)	PS 0–1 (*n* = 39)	PS 2–3 (*n* = 7)
**Disease setting**					
*De novo* metastatic	32/46 (69.6%)	16/22 (72.7%)*^1^	16/24 (66.7%)	25/39 (64.1%)	7/7 (100.0%)
During adjuvant endocrine therapy	10/46 (21.7%)	4/22 (18.2%)	6/24 (25.0%)	10/39 (25.6%)	0 (0.0%)
Recurrence not during endocrine therapy	4/46 (8.7%)	2/22 (9.1%)	2/24 (8.3%)	4/39 (10.3%)	0 (0.0%)
**Concomitant endocrine therapy**					
Letrozole	30/46 (65.2%)	15/22 (68.2%)	15/24 (62.5%)	24/39 (61.5%)	6/7 (85.7%)
Fulvestrant	14/46 (30.4%)	5/22 (22.7%)	9/24 (37.5%)	13/39 (33.3%)	1/7 (14.3%)
**Concomitant endocrine therapy**					
Anastrozole	2/46 (4.3%)	2/22 (9.1%)	0 (0.0%)	2/39 (5.1%)	0 (0.0%)
**Age group (Years)**					
<60	12/46 (26.1%)	–	–	8/39 (20.5%)	4/7 (57.1%)
60–69	10/46 (21.7%)	–	–	9/39 (23.1%)	1/7 (14.2%)
70–79	12/46 (26.1%)	–	–	10/39 (25.6%)	2/7 (28.5%)
≥80	12/46 (26.1%)	–	–	12/39 (30.7%)	–
**Performance status**					
PS 0	18/46 (39.1%)	9/22 (40.9%)	9/24 (37.5%)	–	–
PS 1	21/46 (45.7%)	8/22 (36.4%)	13/24 (54.2%)	–	–
PS 2	4/46 (8.7%)	2/22 (9.1%)	2/24 (8.3%)	–	–
PS 3	3/46 (6.5%)	3/22 (13.6%)	0 (0.0%)	–	–
**Metastatic site**					
Bone	27/46 (58.7%)	14/22 (63.6%)	13/24 (54.2%)	23/39 (59.0%)	4/7 (57.1%)
Lung	18/46 (39.1%)	11/22 (50.0%)	7/24 (29.2%)	16/39 (41.0%)	2/7 (28.6%)
Lymph nodes	18/46 (39.1%)	7/22 (31.8%)	11/24 (45.8%)	15/39 (38.5%)	3/7 (42.9%)
Liver	6/46 (13.0%)	4/22 (18.2%)	2/24 (8.3%)	5/39 (12.8%)	1/7 (14.3%)
Pleural effusion	5/46 (10.9%)	4/22 (18.2%)	1/24 (4.2%)	3/39 (7.7%)	2/7 (28.6%)
Peritoneum	2/46 (4.3%)	1/22 (4.5%)	1/24 (4.2%)	0 (0.0%)	2/7 (28.6%)*^2^

Note: PS, performance status; –, Not applicable; Fisher’s exact test: ^*1^: *p* = 0.003, ^*2^: *p* = 0.020.

Overall, 32 cases (69.6%) presented with new metastatic disease, indicating that the majority were diagnosed with advanced disease at their initial visit. In contrast, 10 patients (21.7%) presented with recurrence during adjuvant endocrine therapy. Regarding endocrine therapy during palbociclib administration, the most frequently prescribed concomitant agent was letrozole (65.2%), followed by fulvestrant (30.4%), while anastrozole was used in only a small number (4.3%).

Regarding age distribution, the cohort was balanced with 22 patients (47.8%) under 70 years old and 24 patients (52.2%) aged 70 years or older. Within the older patient group, 12 patients were aged 70–79 years and 12 patients were aged 80 years or older, revealing a substantial inclusion of very elderly patients—a group often underrepresented in clinical trials—in this real-world cohort. In comparison by age group, *de novo* metastatic disease was found to be significantly more frequent in patients aged <70 years (*p* = 0.003). No other variables showed significant differences between the two age groups. In the comparison by performance status (PS), peritoneal metastasis was significantly more common in patients with PS 2–3 (*p* = 0.020). Additionally, *de novo* metastatic disease tended to be more frequent in the PS 2–3 group, although the difference did not reach statistical significance (*p* = 0.083).

Fulvestrant was selected more frequently in the older group (37.5% vs. 22.7% in the younger group). This is because fulvestrant was often chosen as the first-line agent in cases of recurrence during postoperative endocrine therapy. However, fulvestrant was also selected at the physician’s discretion in some *de novo* patients without prior endocrine therapy.

PS at treatment initiation was generally favorable, with 18 cases (39.1%) classified as PS 0 and 21 cases (45.7%) as PS 1. However, 7 patients (15.2%) had poor PS (2–3), constituting a clinically challenging subgroup. This group showed a markedly higher prevalence of new metastatic disease (100% in PS 2–3 vs. 64.1% in PS 0–1), suggesting that worsening functional status may be closely associated with more aggressive disease at diagnosis. Furthermore, a higher proportion of PS 2–3 patients were under 70 years old (71.4%), whereas the majority of PS 0–1 patients were elderly. This suggests that the deterioration in PS observed in this cohort may reflect disease burden rather than simply the effects of aging.

Regarding metastatic patterns, bone metastases were the most frequent (58.7%), followed by lung metastases (39.1%) and lymph node metastases (39.1%). Liver metastases were relatively uncommon (13.0%), and serosal metastases were relatively rare, with pleural effusion in 10.9% and peritoneal metastases in 4.3%. Subgroup analysis revealed subtle but clinically meaningful differences: pleural effusion and peritoneal seeding were disproportionately high in the PS 2–3 group (28.6% each vs. 7.7% and 0.0% in the PS 0–1 group), suggesting serosal metastases may contribute to functional decline.

This real-world clinical cohort included individuals aged 70 years or older, who often constitute half of the population and are frequently excluded from clinical trials. This real-world clinical cohort included individuals aged 70 years or older, who often constitute half of the population and are frequently excluded from clinical trials. The group with poor performance status comprised only 7 cases, a small number. As this is a real-world clinical report, it is important to note that further investigation with a larger number of cases is necessary.

### 3.2 Treatment Exposure and Modifications

The median treatment duration was 19.9 months. At the data cutoff, 16 patients (34.8%) remained on therapy, whereas 30 (65.2%) had discontinued treatment. The predominant reason for discontinuation was disease progression, which occurred in 24 patients (52.2%). Treatment was discontinued due to toxicity in 2 patients (4.3%) and by patient choice, including difficulty attending the hospital, in 2 patients (4.3%) (Table 2).

**Table 2:** Treatment status and reasons for discontinuation

Group	*n*	Ongoing	Discontinued	Progression	Toxicity	Patient choice
**All**	46	16/46 (34.8%)	30/46 (65.2%)	24/46 (52.2%)	2/46 (4.3%)	2/46 (4.3%)
**Age < 70**	22	8/22 (36.4%)	14/22 (63.6%)	13/22 (59.1%)	1/22 (4.5%)	0/22 (0.0%)
**Age ≥ 70**	24	8/24 (33.3%)	16/24 (66.7%)	11/24 (45.8%)	1/24 (4.2%)	2/24 (8.3%)
**PS 0–1**	39	14/39 (35.9%)	25/39 (64.1%)	20/39 (51.3%)	2/39 (5.1%)	2/39 (5.1%)
**PS 2–3**	7	2/7 (28.6%)	5/7 (71.4%)	4/7 (57.1%)	0/7 (0.0%)	0/7 (0.0%)

Note: “Values are presented as number of patients (%)”. CR, Complete Response, PR, Partial Response, SD, Stable Disease, and PD, Progressive Disease were defined according to RECIST version 1.1. ORR (overall response rate) was calculated as CR + PR, and DCR (disease control rate) as CR + PR + SD. *p*-values represent comparisons between age groups (<70 vs. ≥70 years) and PS groups (PS 0–1 vs. PS 2–3), analyzed using Fisher’s exact test due to small number in several categories.

Dose modifications were common, with 22 patients (47.8%) requiring dose reductions. Among them, 17 (36.9%) were reduced to 100 mg and 5 (10.9%) to 75 mg. Temporary interruptions were observed in 25 patients (54.3%).

Treatment response outcomes are summarized in Table 3. For the entire cohort, the ORR was 60.9% and the DCR was 93.5%. Stratified by age, ORR was 63.6% in patients younger than 70 years and 58.3% in those aged 70 years or older, while DCR was 90.9% and 95.8%, respectively. No statistically significant differences were observed between age groups (ORR, *p* = 0.769; DCR, *p* = 0.600). According to PS, ORR was 59.0% in patients with PS 0–1 and 71.4% in those with PS 2–3, whereas DCR was 92.3% and 100%, respectively, again with no significant differences (*p* = 0.688 and *p* > 0.999, respectively).

**Table 3:** Treatment response in all patients, by age, and by performance status

Response	All (*n* = 46)	<70 yrs (*n* = 22)	≥70 yrs (*n* = 24)	*p* (Age)	PS 0–1 (*n* = 39)	PS 2–3 (*n* = 7)	*p* (PS)
CR	6/46 (13.0%)	4/22 (18.2%)	2/24 (8.3%)	0.405	4/39 (10.3%)	2/7 (28.6%)	0.221
PR	22/46 (47.8%)	10/22 (45.5%)	12/24 (50.0%)	0.777	19/39 (48.7%)	3/7 (42.9%)	>0.999
SD	15/46 (32.6%)	6/22 (27.3%)	9/24 (37.5%)	0.539	13/39 (33.3%)	2/7 (28.6%)	>0.999
PD	3/46 (6.5%)	2/22 (9.1%)	1/24 (4.2%)	0.600	3/39 (7.7%)	0/7 (0.0%)	>0.999
**ORR**	28/46 (60.9%)	14/22 (63.6%)	14/24 (58.3%)	0.769	23/39 (59.0%)	5/7 (71.4%)	0.688
**DCR**	43/46 (93.5%)	20/22 (90.9%)	23/24 (95.8%)	0.600	36/39 (92.3%)	7/7 (100.0%)	>0.999

Note: PS, Performance Status; CR, Complete Response; PR, Partial Response; SD, Stable Disease; PD, Progressive Disease; ORR (overall response rate) = CR + PR; DCR (disease control rate) = CR + PR + SD; *p*-values were calculated using Fisher’s exact test.

Notably, even patients with poor PS 2–3 achieved a high DCR, comparable to those with PS 0–1. Similarly, elderly patients (≥70 years) demonstrated response rates equivalent to younger patients, indicating that both subgroups could benefit from treatment with palbociclib plus endocrine therapy.

Differences in treatment efficacy between metastatic sites are shown in Table 4. The ORR ranged from 48.1% (bone metastases) to 83.3% (serosal metastases), while the DCR ranged from 88.9% to 100%.

**Table 4:** Overall response rate (ORR) and Disease control rate (DCR) by metastatic site

Metastatic site	*n*	ORR (%)	DCR (%)
Lung	18	12/18 (66.7%)	16/18 (88.9%)
Lymph nodes	18	13/18 (72.2%)	16/18 (88.9%)
Liver	6	3/6 (50.0%)	6/6 (100.0%)
Bone	27	13/27 (48.1%)	26/27 (96.3%)
Serosal (peritoneum and/or pleural effusion)	6	5/6 (83.3%)	6/6 (100.0%)
Chi-square test		*p* = 0.326	*p* = 0.666

Note: CR, Complete Response; PR, Partial Response; SD, Stable Disease; PD, Progressive Disease; ORR (overall response rate) = CR + PR; DCR (disease control rate) = CR + PR + SD.

Although both ORR and DCR tended to be higher in patients with serosal (pleural and/or peritoneal) metastases, no statistically significant differences were observed among metastatic sites (ORR, *p* = 0.326; DCR, *p* = 0.666). Given the limited number of patients in each subgroup, additional studies with larger cohorts are needed to clarify potential site-specific differences in treatment response.

### 3.3 Efficacy Outcomes

The median PFS for the entire cohort was 26.6 months (range, 1.4–69.5).

**Figure 1:**
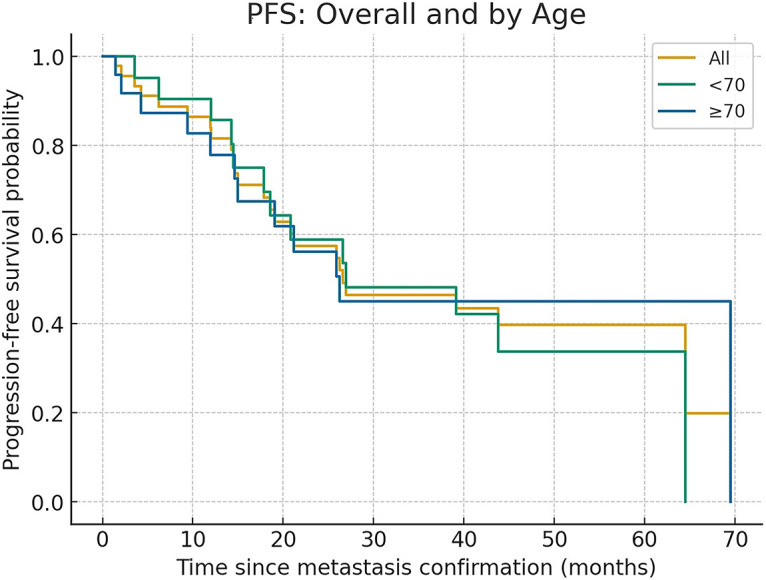
PFS for all cases and by age group

When stratified by age, the median PFS was 26.9 months (range, 2.0–64.5) in patients younger than 70 years and 26.2 months (range, 1.4–69.5) in those aged 70 years or older, with no significant difference between the two groups (log-rank *p* = 0.76; Cox univariate HR 0.89, 95% CI 0.41–1.96, *p* = 0.78) (Fig. 1).

**Figure 2:**
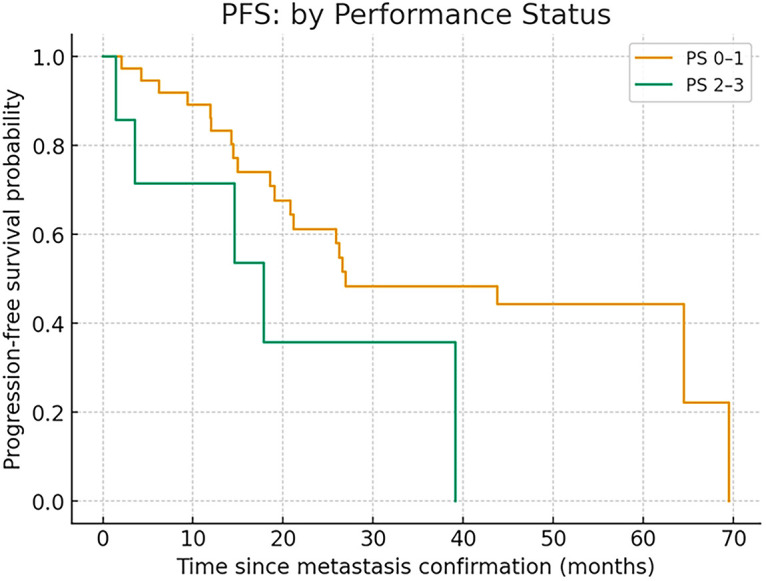
PFS in PS 0–1 and PS 2–3

When stratified by performance status (PS), the median PFS was 26.9 months (range, 2.0–69.5) in patients with PS 0–1 and 17.8 months in those with PS 2–3, showing numerically shorter PFS in the latter group, although the difference did not reach statistical significance (log-rank *p* = 0.099) (Fig. 2).

In the Cox proportional hazards model, PS 2–3 tended to be associated with shorter PFS in the univariate analysis (HR 2.65, 95% CI 0.96–7.35, *p* = 0.06).

In the multivariate model adjusted for age and serosal involvement (pleural effusion or peritoneal dissemination), PS 2–3 remained associated with shorter PFS (HR 3.57, 95% CI 1.10–11.58, *p* = 0.03).

Although these findings suggest that patients with poor performance status and serosal involvement may experience shorter PFS, the results should be interpreted with caution because of the limited sample size.

In Cox proportional hazards analysis, age ≥70 years was not associated with shorter PFS (HR 0.89, 95% CI 0.41–1.96, *p* = 0.78).

PS 2–3 tended to show shorter PFS and reached significance in one adjusted model (HR 3.57, 95% CI 1.10–11.58, *p* = 0.03), although interpretation should be cautious given the limited sample size.

### 3.4 Safety Outcomes

Adverse events observed in this cohort are summarized in Table 5. The most frequent hematologic toxicities were leukopenia and neutropenia. Leukopenia was documented in 40 patients (86.7%), with grade 1 in 14 (30.4%), grade 2 in 16 (34.8%), and grade ≥ 3 in 10 (21.7%). Neutropenia occurred in 37 patients (80.4%), including grade 1 in 11 (23.9%), grade 2 in 13 (28.3%), and grade ≥ 3 in 13 (28.3%). Anemia was less common, observed in 12 patients (26.1%), and grade ≥ 3 in only one case (2.2%). Thrombocytopenia was rare, detected in four patients (8.7%), with grade ≥ 3 in a single patient.

**Table 5:** Adverse events by grade (All patients, *n* = 46)

Adverse event	All (*n* = 46)	Grade 1	Grade 2	Grade ≥ 3
Leukopenia	40 (86.7%)	14 (30.4%)	16 (34.8%)	10 (21.7%)
Neutropenia	37 (80.4%)	11 (23.9%)	13 (28.3%)	13 (28.3%)
Anemia	12 (26.1%)	9 (19.6%)	2 (4.3%)	1 (2.2%)
Stomatitis	7 (15.2%)	7 (15.2%)	0	0
Constipation	5 (10.8%)	5 (10.9%)	0	0
Rash	5 (10.8%)	4 (8.7%)	1 (2.2%)	0
Thrombocytopenia	4 (8.7%)	2 (4.3%)	1 (2.2%)	1 (2.2%)
Liver dysfunction	4 (8.7%)	4 (8.7%)	0	0
Fatigue	3 (6.5%)	3 (6.5%)	0	0
Arthralgia	2 (4.3%)	2 (4.3%)	0	0
Dysgeusia	2 (4.3%)	2 (4.3%)	0	0
Abdominal pain	2 (4.3%)	1 (2.2%)	1 (2.2%)	0
Pruritus	2 (4.3%)	2 (4.3%)	0	0
Diarrhea	2 (4.3%)	2 (4.3%)	0	0
Loss of appetite	1 (2.2%)	1 (2.2%)	0	0
Nausea	1 (2.2%)	1 (2.2%)	0	0
Peripheral neuropathy	1 (2.2%)	1 (2.2%)	0	0
Interstitial lung disease	0	0	0	0
Edema	0	0	0	0
Heart failure	0	0	0	0

Non-hematologic adverse events were generally mild. Stomatitis (15.2%), constipation (10.9%), and rash (10.9%) were the most frequent, all limited to grade 1–2 severity. Fatigue was recorded in three patients (6.5%), exclusively grade 1. Other events such as dysgeusia, abdominal pain, pruritus, diarrhea, loss of appetite, nausea, and peripheral neuropathy were infrequent, each occurring in fewer than 5% of patients and confined to grade 1–2. No cases of interstitial lung disease, edema, or heart failure were observed (Table 5).

Subgroup analyses according to age revealed similar overall toxicity patterns between patients aged <70 years and those aged ≥70 years. Hematologic toxicities, including neutropenia and leukopenia, remained the most common adverse events across both age groups. However, anemia appeared more frequent in elderly patients (≥70 years: 29.2% vs. <70 years: 9.1%), and one case of grade ≥ 3 anemia was observed only in the older cohort. Although the difference did not reach statistical significance, this trend suggests that elderly patients may be more susceptible to treatment-related anemia and may require closer monitoring of hemoglobin levels during therapy. Anemia improved to Grade 1 in all cases following palbociclib dose reduction and treatment interruption. In contrast, the incidence of non-hematologic toxicities, including gastrointestinal or dermatologic events, was generally low and comparable between age groups, with few severe cases observed (Table 6).

**Table 6:** Adverse events by grade and age group

Adverse event	<70 yrs (*n* = 22)			≥70 yrs (*n* = 24)		
	Grade 1	Grade 2	Grade ≥ 3	Grade 1	Grade 2	Grade ≥ 3
Leukopenia	7/22 (31.8%)	8/22 (36.4%)	4/22 (18.2%)	7/24 (29.2%)	8/24 (33.3%)	6/24 (25.0%)
Neutropenia	5/22 (22.7%)	8/22 (36.4%)	5/22 (22.7%)	6/24 (25.0%)	5/24 (20.8%)	8/24 (33.3%)
Liver dysfunction	3/22 (13.6%)	0	0	1/24 (4.2%)	0	0
Constipation	3/22 (13.6%)	0	0	2/24 (8.3%)	0	0
Stomatitis	3/22 (13.6%)	0	0	4/24 (16.7%)	0	0
Anemia	2/22 (9.1%)	0	0	7/24 (29.2%)	2/24 (8.3%)	1/24 (4.2%)
Fatigue	2/22 (9.1%)	0	0	1/24 (4.2%)	0	0
Thrombocytopenia	1/22 (4.5%)	0	0	1/24 (4.2%)	1/24 (4.2%)	1/24 (4.2%)
Nausea	1/22 (4.5%)	0	0	0	0	0
Diarrhea	1/22 (4.5%)	0	0	1/24 (4.2%)	0	0
Pruritus	1/22 (4.5%)	0	0	1/24 (4.2%)	0	0
Rash	1/22 (4.5%)	0	0	3/24 (12.5%)	1/24 (4.2%)	0
Dysgeusia	1/22 (4.5%)	0	0	1/24 (4.2%)	0	0
Arthralgia	1/22 (4.5%)	0	0	1/24 (4.2%)	0	0
Abdominal pain	0	0	0	1/24 (4.2%)	1/24 (4.2%)	0
Peripheral neuropathy	0	0	0	1/24 (4.2%)	0	0
Loss of appetite	0	0	0	1/24 (4.2%)	0	0
Interstitial lung disease	0	0	0	0	0	0
Heart failure	0	0	0	0	0	0

Adverse events categorized as PS0–1 and 2–3 are shown in Table 7. Hematologic adverse events such as leukopenia and neutropenia occurred at similar frequencies in both cohorts, with grade ≥ 3 neutropenia observed in 28.2% of PS 0–1 patients and 28.6% of PS 2–3 patients. Likewise, severe leukopenia was documented in 20.5% and 28.6% of patients, respectively. Anemia was relatively common in PS 0–1 patients (23.1%, 2.6% and 2.6% in grades 1, 2, and 3, respectively), whereas in the PS 2–3 group, only a single grade 2 case was reported, without grade ≥ 3 events. Non-hematologic toxicities remained infrequent across both groups; fatigue, diarrhea, pruritus, rash, peripheral neuropathy, loss of appetite, and constipation were observed only in the PS 0–1 group, whereas nausea appeared in PS 2–3 patients. Importantly, no excess risk of severe non-hematologic complications, including interstitial lung disease or cardiac failure, was detected in patients with poor performance status. Collectively, these findings suggest that even patients with PS 2–3 can tolerate treatment without disproportionate increases in toxicity, although careful monitoring remains warranted given the limited sample size. Similarly, patients with poor PS (2–3) experienced comparable rates of hematologic and non-hematologic toxicities to those with PS 0–1, without any excess risk of severe complications (Table 7).

**Table 7:** Adverse events by grade and performance status

Adverse event	PS 0–1 (*n* = 39)			PS 2–3 (*n* = 7)		
	Grade 1	Grade 2	Grade > 3	Grade 1	Grade 2	Grade > 3
Leukopenia	12/39 (30.8%)	13 (33.3%)	8/39 (20.5%)	2/7 (28.6%)	3/7 (42.9%)	2/7 (28.6%)
Neutropenia	9/39 (23.1%)	11 (28.2%)	11/39 (28.2%)	2/7 (28.6%)	2/7 (28.6%)	2/7 (28.6%)
Liver dysfunction	3/39 (7.7%)	0	0	1/7 (14.3%)	0	0
Constipation	5/39 (12.8%)	0	0	0	0	0
Stomatitis	6/39 (15.4%)	0	0	1/7 (14.3%)	0	0
Anemia	9/39 (23.1%)	1 (2.6%)	1/39 (2.6%)	0	1/7 (14.3%)	0
Fatigue	3/39 (7.7%)	0	0	0	0	0
Thrombocytopenia	2/39 (5.1%)	1/39 (2.6%)	0	0	0	0
Nausea	0	0	0	1/7 (14.3%)	0	0
Diarrhea	2/39 (5.1%)	0	0	0	0	0
Pruritus	2/39 (5.1%)	0	0	0	0	0
Rash	4/39 (10.3%)	1/39 (2.6%)	0	0	0	0
Dysgeusia	1/39 (2.6%)	0	0	1/7 (14.3%)	0	0
Arthralgia	1/39 (2.6%)	0	0	1/7 (14.3%)	0	0
Abdominal pain	0	1/39 (2.6%)	0	1/7 (14.3%)	0	0
Peripheral neuropathy	1/39 (2.6%)	0	0	0	0	0
Loss of appetite	1/39 (2.6%)	0	0	0	0	0
Interstitial lung disease	0	0	0	0	0	0
Heart failure	0	0	0	0	0	0

Overall, hematologic toxicities, particularly neutropenia and leukopenia, represented the main safety concern; however, the majority of non-hematologic events were mild and manageable. Importantly, the absence of excess toxicity in elderly patients or those with poor PS suggests that palbociclib plus ET can be administered safely across a broad spectrum of real-world patients, consistent with findings from pivotal clinical trials.

## 4 Discussion

This real-world study provides important insights into the clinical use of palbociclib plus ET for patients with recurrent or *de novo* HR+/HER2− breast cancer in Japan. By including a substantial proportion of elderly patients and those with impaired PS, our analysis addresses a gap in evidence left by pivotal clinical trials that underrepresented these populations. The findings demonstrate that palbociclib-based therapy achieves durable efficacy and has a manageable safety profile across a wide spectrum of patients, supporting its generalizability to daily practice.

Our cohort achieved a median PFS of 26.6 months, which is slightly longer than the 24.8 months reported in PALOMA-2 for palbociclib plus letrozole [3]. This also compares favorably with MONALEESA-2 (25.3 months with ribociclib) [6] and MONARCH-2 (16.4 months with abemaciclib plus fulvestrant) [9]. Importantly, the ORR of 60.9% and DCR of 93.5% in our study mirror those of pivotal trials, reinforcing the external validity of these results.

Kaplan–Meier curves confirmed no survival disadvantage in patients aged 70 or older, while PS 2–3 patients had numerically shorter PFS. These observations highlight that clinical benefit can be extended to broader populations, although functional status remains an important prognostic factor.

The absence of significant PFS differences between younger (<70 years, 26.9 months) and older patients (≥70 years, 26.2 months; *p* = 0.760) underscores that chronological age does not diminish treatment benefit. These findings confirm pooled analyses from the PALOMA trial [11,12] and real-world evidence from Western cohorts [13–16], which consistently reported comparable outcomes in elderly patients. Our results extend this evidence to Japanese practice, and importantly, include patients aged ≥80 years, who are often underrepresented in trials.

In contrast, PS-stratified outcomes revealed a clinically relevant difference: patients with PS 0–1 achieved a median PFS of 26.9 months, compared with 17.8 months in those with PS 2–3 (*p* = 0.099). While this did not reach statistical significance due to small numbers, it suggests that functional impairment remains a critical determinant of prognosis. Notably, all PS 2–3 patients achieved disease control, indicating that stabilization of tumor burden is achievable even in this challenging group. PS2–3 cases are limited to seven, requiring careful judgment and further investigation with a larger number of cases.

The safety profile was dominated by hematologic adverse events, with leukopenia (65.2%) and neutropenia (52.2%; grade ≥ 3 in 28.3%) being the most common. These rates are in line with PALOMA-2 [3]. Anemia was observed in 26.1% of patients, with only 2.2% experiencing grade ≥ 3 events. Elderly patients showed a tendency toward higher anemia rates, consistent with prior Japanese reports [17,18]. Non-hematologic toxicities, including stomatitis, constipation, and rash, were generally mild and manageable.

Dose reductions occurred in 47.8% of patients, mostly to 100 mg. Importantly, efficacy was maintained despite these modifications, echoing evidence from Layman et al. (2025) that dose reductions do not compromise outcomes [14]. This highlights the role of dose flexibility as a supportive strategy rather than a therapeutic compromise.

Our data strongly support the safety of palbociclib in elderly populations. Median PFS in patients aged 70 or older was virtually identical to that of younger patients. Japanese studies by Iwata (2018) [17] and Sawaki et al. (2022) [18] also reported preserved efficacy in elderly cohorts, despite somewhat higher rates of hematologic toxicity. Together with Canadian [16] and European [15] cohorts, the evidence indicates that age alone should not preclude treatment. Current guidelines—including European Society for Medical Oncology (ESMO) [26], National Comprehensive Cancer Network (NCCN) [21], American Society of Clinical Oncology (ASCO) [22], and The Japanese Breast Cancer Society (JBCS) [27]—all recommend that treatment decisions be based on fitness and comorbidities rather than chronological age, a principle reaffirmed by our results.

Seven patients (15.2%) in our study had PS 2–3. Their median PFS of 17.8 months was shorter than that of PS 0–1 patients, but all achieved disease control. These findings highlight that careful patient selection can extend the benefit even to those with functional impairment. Importantly, poor PS in this cohort was strongly associated with *de novo* metastatic disease and serosal involvement, suggesting that tumor burden rather than comorbidity was the main driver of functional decline.

Patients whose PS deterioration stems from tumor burden may recover function with therapy, while those impaired by comorbidities may face excess toxicity. This distinction is critical. Similar conclusions were drawn by Porte et al. (2020) [28] and Sobrini-Morillo et al. (2024) [29]. Thus, physicians should avoid automatically excluding PS 2–3 patients and instead assess the underlying cause of impairment.

Our findings align with global real-world evidence. Brufsky et al. (2021) [13] and Layman et al. (2025) [14] demonstrated similar outcomes in the United States, while Mycock et al. (2022) [16] confirmed efficacy in Canadian practice. European cohorts such as Brain et al. (2024) [15] and Diéras et al. (2019) [30] also reported preserved benefit in routine care.

In Asia, Iwata (2018) [17] and Sawaki et al. (2022) [18] established palbociclib’s utility in Japanese patients, Kim et al. (2021) [19] in Korean patients, Xu et al. (2021) [20] in pooled Asian analyses, and Zhu et al. (2023) [31] in Chinese patients. Together, these findings underscore the reproducibility of palbociclib outcomes across ethnic groups and healthcare systems.

Although our retrospective study did not capture quality-of-life (QOL) or patient-reported outcomes (PROs), prior evidence suggests that CDK4/6 inhibitors do not compromise QOL. PALOMA-2 demonstrated maintenance of QOL and delayed symptom deterioration with palbociclib [32], while MONARCH-2 showed improved pain control with abemaciclib [33]. These data are especially relevant in elderly and frail patients, for whom preservation of daily function and independence is critical.

This study has several limitations. First, it was a single-center retrospective study with a relatively small sample size, which may limit the generalizability of the findings. Second, the follow-up period was insufficient to assess long-term outcomes such as overall survival. Third, patient selection bias cannot be excluded, as treatment decisions and dose modifications were at the physicians’ discretion. Finally, quality-of-life and patient-reported outcomes were not evaluated, which are particularly relevant in elderly and poor-performance-status populations. Therefore, larger multicenter prospective studies are warranted to validate these findings.

## 5 Conclusion

In conclusion, palbociclib plus ET achieved prolonged PFS of 26.6 months, high response rates, and manageable safety in this Japanese real-world cohort. Elderly patients and selected PS 2–3 patients benefited meaningfully, supporting the principle that neither age nor PS should serve as exclusion criteria. With appropriate monitoring and dose adjustments, palbociclib can be safely extended to vulnerable populations, ensuring equitable access to effective therapy.

## Data Availability

The data presented in this study are available on request from the corresponding author.

## Abbreviations

CDK4/6 Inhibitors: Cyclin-Dependent Kinase 4/6 Inhibitors
ET: Endocrine therapy
HR: Hormone receptor
HER2: Human epidermal growth factor receptor 2
PS: Performance status
PFS: Progression-free survival
ORR: Overall response rate
DCR: Disease control rate
RWE: Real-world evidence
AEs: Adverse events
GnRH: Gonadotropin-releasing hormone
QOL: Quality-of-life
